# Characterizing intercampus migration in a private university of Baja California, Mexico

**DOI:** 10.12688/f1000research.111079.2

**Published:** 2023-03-21

**Authors:** Lucia Beltrán, Damián-Emilio Gibaja-Romero, Rosa-María Cantón-Croda

**Affiliations:** 1 CETYS Universidad, Ensenada, Baja California, 22000, Mexico; 2 Area de Matematicas, UPAEP-University, Puebla, Puebla, 72410, Mexico; 3 Decanato de Ingenierias, UPAEP-University, Puebla, Puebla, 72410, Mexico

**Keywords:** University dropout, predictive modeling, data mining, intercampus migration

## Abstract

Given the socioeconomic and environmental differences between Mexico’s geographical regions, having a multi-campus system is common for private and public universities. Hence, students may choose to migrate from one campus to another. Although such a phenomenon is not properly students’ desertion, students’ migration impacts campus’ main indicators: enrollment growth goals, terminal efficiency, accreditation programs, and revenue. Thus, the campus of origin internalizes migration as students’ desertion. By considering a campus from a private multi-campus university in Baja California, Mexico, this study characterizes and predicts students’ migration and predicts by determining the socioeconomic and academic variables that impact the probability of moving to a different campus. Our database comprises quantitative and qualitative information of 356 dropout students from 2008 to 2018. Hence, we apply the logistic regression technique to build a predictive model; we found that the most significant predictive variables are the GPA results, age, financial support, and academic development. So, our main results characterize migrant students as having top grades, coming from the high school campus, and attending engineering programs. Surprisingly, economic variables are not significant in choosing to migrate from one campus to another.

## Introduction

In 2020, the Organisation for Economic Co-operation and Development (OECD) estimated that 40% of the worldwide population between the ages 25 and 64 had a bachelor’s degree. The country with the highest coverage was Canada, with 60% of its people with a bachelor’s degree, and China had the lowest percentage with 10% of its adult population. Concerning Mexico, the OECD estimated that 19% of the Mexican people have a bachelor’s degree, a lower percentage in comparison with similar economies such as Brazil (25%), Chile (20%), and Colombia (20%). It is worth recalling that this indicator measures human capital, signals individual skills, and generates positive externalities concerning social and economic outcomes for countries ( Education at a Glance 2021: OECD Indicators, 2021). Thus, preventing total or partial students’ dropout is crucial for private and public universities due to the importance of higher education in a developing country such as Mexico.

Although Mexico has increased higher education coverage since 1950 in its territory, going from one to 36 million students ( OECD, 2019a), the dropout rate has also increased in the last ten years from 7% to 8.2% ( INEGI, 2021). In the case of the Baja California state, its dropout rate significantly increased from 1.1 to 6.1 between 2005 and 2021. To prevent such a phenomenon, the Federal Ministery of Education (SEP by its acronym in Spanish) analyzes students’ success by monitoring indicators related to failure, abandonment or total desertion, and terminal efficiency.

In this work, we focus on intercampus migration since the change in enrollment impacts the original campus in the growth goals of its student population, the finances due to the reduction of enrollment income, and the accreditation processes for academic programs on campus as terminal efficiency is reduced. So, even though migrant students remain in the same educational system, campuses internalize such a phenomenon as school dropout, which is of interest for Mexican private universities since they commonly have a multi-campus structure due to the extension of the Mexican territory. Moreover, moving from one campus to another does not avoid students’ desertion ( Veloso & Rodríguez-Gómez, 2020).

The present study is quantitative, non-experimental, and longitudinal with a descriptive intention to identify the variables that affect inter-camp migration. Hence, this paper characterizes and predicts the students who migrate to other campuses, which is helpful for designer retention strategies and improving processes. We identify the academic, personal, and campus factors that characterize migrant students through a binary logistic regression. The analysis that we develop aims to construct a better predictive model that allows the design of early intervention strategies since intercampus migration implies desertion and loss of competitiveness in the origin campuses.

Tinto (1989) emphasizes the variability of school dropouts by mentioning that not all dropouts require an institutional intervention since the character and the causes define the type of abandonment. Moreover, abandonment depends on the perspective that we use to study it. For example, financial and competitiveness approaches differently interpret such a phenomenon. Even more, the abandonment definition relies on institutional, social, or personal objectives ( Claudia & Santelices, 2020).

Formally, total dropout is “the percentage of students who drop out of school activities during the school year (extracurricular dropouts) and at the end of it (inter curricular dropouts) concerning the total number of students enrolled in the school year” ( Tamez *et al.*, 2006). School dropout in universities is an issue that has been analyzed considering the academic record ( Eckert & Suénaga, 2015), the academic performance of students ( Bernardo *et al.*, 2016), the economic situation of families ( Ramírez & Grandón, 2018), the structures and policies of the Institutions ( Vera Noriega *et al.*, 2012), the opportunities to thrive in the academic program ( Hernández *et al.*, 2016), among others.

In Mexico, public education is managed by the federal and state governments. While superior education follows a rigorous selection process, its supply is insufficient. Thus, private universities arise from the need to solve the low admission capacity of public universities ( Chen, Xu, Mo, and Bian, 2018). However, private universities also have selection processes based on the academic, social, financial, and personal attributes of prospects to guarantee that their students have the competencies required to fulfill their academic plan, graduation requirements, and efficiency. So, selection processes are necessary to ensure the quality standards set by the Institution ( Zozaya, 2013). According to the 2019-2020 School Cycle Student Statistics summary, published by the Ministry of Public Education (SEP), the total number of undergraduate students was 3,813,626 students, with 40% of them enrolled in the private System ( Secretary of Public Education, 2020).

As far as we know, students’ migration appears as a Mexican phenomenon since the growth of Mexican private universities is based on increasing their enrollment by building new campuses. However, in recent years, top universities like NYU and Texas A&M have established branch campuses in the United Arab Emirates and Singapore. So, our paper is closely related to the literature that analyzes the competition between the home and the branch campuses ( Kosmützky, 2018). Our main contribution relies on identifying those features that promote students’ migration from one campus to another, which may identify those factors that impact the recruitment of students for international branch campuses ( Wilkins & Huisman, 2011).

The article is structured in the following sections. The second section presents a literature review concerning students dropping out. Next, section three describes the database we use and the mathematical model we apply to understand the factors that impact student migration. Sections four and five present and discuss the main results, respectively. The final section describes the conclusions of our work.

## Literature review

### Theoretical references of university dropout

One of the most relevant theoretical models of school dropout is Tinto (1975), which indicates that dropout is related to race, gender, ability, social status, previous academic experiences, and residency. In addition, Tinto emphasizes variables that represent motivation, such as the expectations of the chosen career and resilience to achieve the academic degree. The previous factors indicate the students’ commitment to their objectives, which, according to Tinto, can be decisive in their decision to drop out. Tinto’s model serves as the basis and is complemented by the theories of Pascarella & Terenzini (1980); Ethington (1990); Bean (1985); Spady (1970), and Astin (1999) on the existence of exogenous factors with the ability to influence dropouts. For example, financial factors and the perception of benefits (student investment against what he receives).

Bean and Metzner (1985) emphasize the academic, social, psychological, and environmental factors at the time of entering the university as variables that define the student’s academic performance in his career. Cabrera, Nora, and Castaneda (1993) integrate some variables from the Tinto (1975) and Bean and Metzner (1985) models in a longitudinal study during the first year of study at an academic institution in Australia. Their model emphasizes the need for students to receive financial support and the cost-benefit ratio since these variables directly influence the decision to dropout from school.

Concerning private institutions, the student’s perception (what they pay for) plays a crucial role in school dropout. So, desertion is affected by the financial support system, prestige, educational quality, and university faculty ( Cabrera *et al.*, 1993; Pascarella and Terenzini, 1980; Ethington, 1990).

### Some case studies of dropout and migration

The literature on student dropout seeks to reduce it and increase terminal efficiency. Analyzing different variables, such as self-efficacy ( Charris *et al.*, 2017), is sought to find a correlation between personal attributes and the students’ permanence. For example, Navarro *et al.* (2017) studied a sample of 322 students from the 2015-1 period, male and female, active and dropouts at a university in Barranquilla, Colombia. Using the General Self-Efficacy Scale (EAG), Navarro *et al.* did not find a significant correlation between efficacy and permanence or a substantial correlation between effectiveness and attrition. However, they find that dropouts increase among students with a job compared to those without jobs. Thus, students who are not full-time have a higher risk of dropping out.

The grade point average (GPA), the years of advancement in the career, and the university selection test score represents students’ academic performance in a university. Ramírez and Grandón (2018) analyze student dropout considering a sample of 5,288 Chilean students belonging to four consecutive student cohorts from 44 university programs. Using decision trees with optimized parameters, they analyze the impact on dropout by considering factors classified in demographics (age and gender), university history (admission exam score, grade point average in the last period), economic (family income level and type of high school), and academic performance (progress, and general grade point average).

Students who move from their places of residence to continue their university studies are also possible causes of desertion. Hernández *et al.* (2016) predict the risk of desertion for 134 students of the academic program “Engineering in Information Technology and Communications” at the Higher Technological Institute of Misantla, Mexico. By applying logistic regression, clusters generation, decision trees, and neural networks, Hernández et al. conclude that the student’s place of origin significantly impacts school dropout. Simillarly, Eckert and Suénaga (2015) characterize students’ attrition for the “Computer Engineering” degree at the Gastón Dachary University in Argentina. By applying the Classifier Algorithm C4.5, Decision Trees, Naïve Bayes Augmented to Tree (TAN), and OneR Rules, the authors find that the place of origin and the number of failed subjects in the first year of studies are factors with a significant impact on desertion.

Regarding the migration or transfer of students between different institutions, which from the student’s perspective is not about dropping out or abandonment but about persistence and change, Veloso and Rodríguez-Gómez (2020) carried out a study in a Chilean university to identify the main factors influencing students’ migration. They find that students move to other institutions due to faculty, personal expectations, and academic failure perceptions, which accumulate until generating a break in the student who chooses to migrate.

At an international level, particularly in the United States, students’ migration to other campuses has to do with transportation and mobility to reach the campus ( Chamely-Wiik *et al.*, 2021). In other words, those who do not live in the campus’ dormitories deal with transport costs, which increase the risk of desertion. Students’ migration is quite natural since Americans face it many times: from community colleges to universities, from a four-year bachelor’s program to two-year bachelor’s program, or from public to private universities ( Ishitani & Flood, 2018). So, this branch of the literature concerns generating adaptation strategies ( Chamely-Wiik *et al.*, 2021) to support and integrate students from other campuses or regions ( York & Fernandez, 2018). For instance, accrediting institutions evaluate the previous programs for students from other countries or regions ( Anwar & Richards, 2018).

### Data mining and supervised learning

The use of data mining facilitates decision-making since it enables new approaches to problem-solving by discovering hidden patterns and relationships in datasets. Hence, data mining allows an inductive approach to decision support systems (Decision Support Systems, DSS). The data mining algorithms are divided into supervised and unsupervised.

The supervised methodologies require a group of previously classified data and knowing the value of the attributes in advance. On the other hand, the supervised learning algorithms use pre-established input variables known as independent variables, which can have a quantitative, qualitative, or categorical value, to predict the importance of the outputs or dependent variables, whose nature can be quantitative or categorical. The fundamental purpose is to identify a pattern to predict the expected response ( Hastie *et al.*, 2013).

The supervised techniques are those where the data does not have any label or classification; that is to say, no categorical or numerical objective value is known a priori ( Gironés *et al.*, 2017). The most widely used learning algorithms are linear predictors since they are easy to interpret. However, these models seek to respond with a single value (linear regression) or a binary classification (logistic regression), among others, by ignoring complex relationships ( Shalev-Shwartz & Ben-David, 2013).

## Methodology

### Ethical approval

The ethical research committee of CETYS University granted retrospective approval for this study and publication of the results, in the resolution D-CEI100 on March 25 ^th^, 2022. Based on Mexican regulations pertaining research for health purposes, the ERC stated that data of the research are anonymous, and there is no need for individual informed consent for this study.

### Setting

Our case study refers to a non-profit private University System located in Baja California, Mexico, which a group of Counselors sponsors. The system has three campuses located in the cities of Mexicali, Tijuana, and Ensenada. The institution’s academic offer concentrates on three levels: high school, professional, and postgraduate ( CETYS University, 2010).

The Ensenada campus is the international campus of the University System. It offers two high school programs, 12 bachelor’s degrees (six from the business and management school and six from the engineering school), five master’s programs, and a doctorate program. It is the smallest campus in the university system, with 1107 students enrolled from August to December 2019. In contrast, the Mexicali and Tijuana campuses have 3724 and 3421 students, respectively ( Cierre de Campaña 2019, CETYS Universidad Campus Ensenada, 2019).

**Figure 1.**
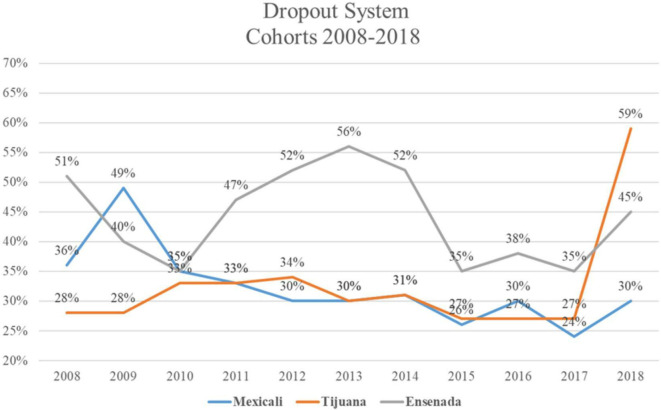
Dropout System Cohorts 4 ^th^ year 2008-2018 ( CETYS University, 2018).

The current dropout rate in Ensenada’s undergraduate programs is 30%, which is not desirable since the institutional goal is 20% ( CETYS University, 2018). Although the Ensenada campus has special financial support ( CETYS Universidad, 2016), its dropout rate is higher than other campuses, except for 2017 and 2018, where the Tijuana campus reports the highest dropout rate, and 2009 where the Mexicali deals with the highest desertion rate, see Figure 1.

The three campuses have the same academic programs, educational model, philosophy, mission, educational principles, and values; the financial support programs also apply to all the campuses. However, the Ensenada Campus has an exclusive scholarship, called PAFENI, that the rectory grants to support high scholars who want to start their undergraduate studies. It is important to note that scholarships can be transferred from one campus to another except for the PAFENI scholarship since such a scholarship pretends to boost enrolling at the Ensenada campus.

**Figure 2.**
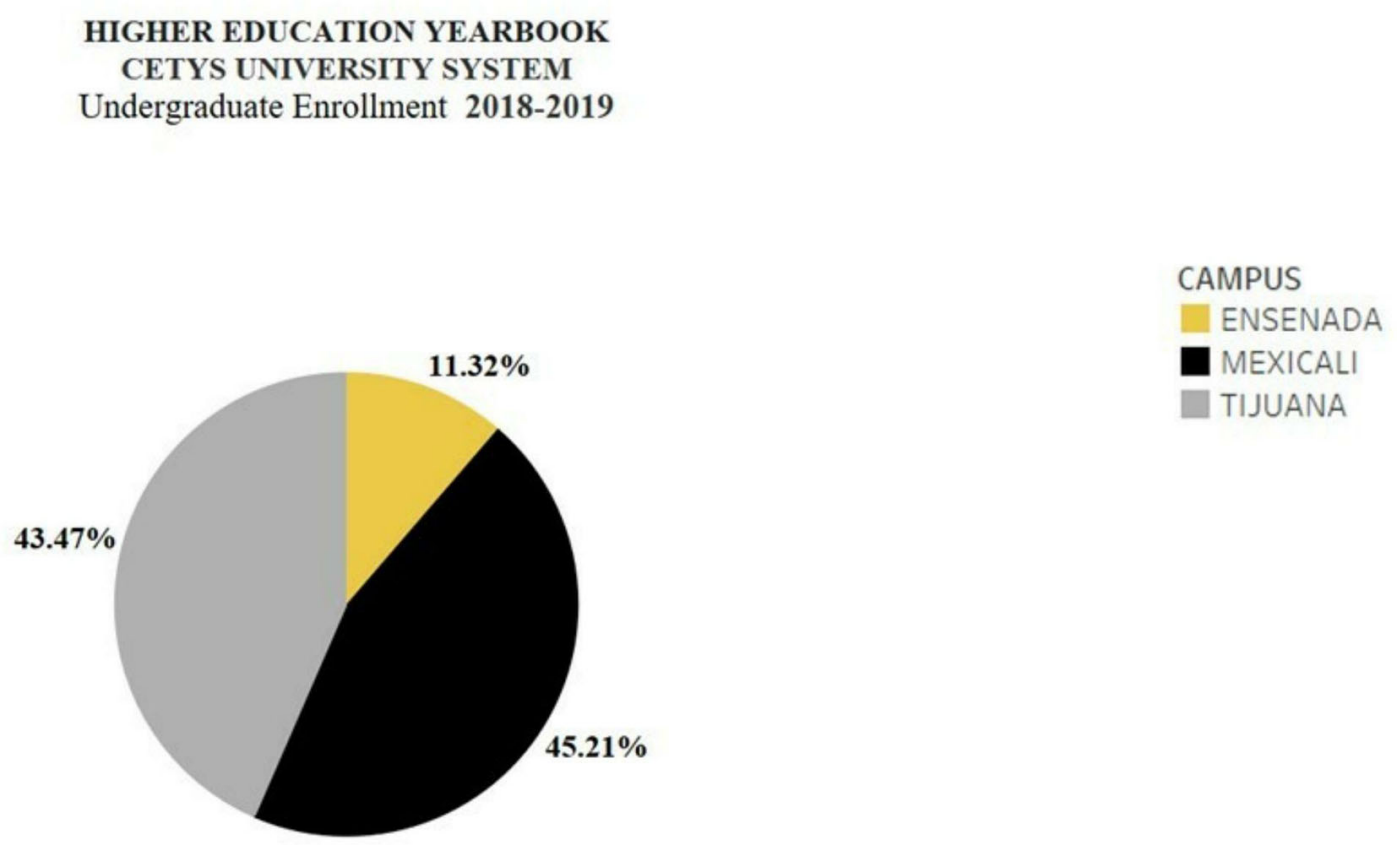
Distribution of undergraduate enrollment in the CETYS University System ( CETYS University, 2019).

The distribution of undergraduate enrollment in the system, as can be seen in Figure 2, is mainly concentrated between the Mexicali campus and the Tijuana campus.

### The data

Our database considers dropout students from all undergraduate programs in the Ensenada Campus between January 2008 and June 2018. The database comprises 356 records, of which 26% relate to campus migration. The data extraction was carried out as follows: first, we identify the current and historical data sources, formal and informal, that contain relevant information to fulfill the purpose of this study.

Consequently, the information generated around the student was gathered, from their first contact with the Ensenada Campus to the moment when they leave the campus without completing their studies. So, the data were obtained from the university’s main digital sources such as promotion, scholar WEB System, finance, student information portal, and the Education Center for Student Development. The data was collected to build a database whose logical model represents the different dimensions that characterize the dropout students. Table 1 shows the database classification. Note that we consider four groups: personal details, entrance to the university, academic program attributes, and institutional experience. We process these data through the R programming tool version 4.1.2, and we use the BigML cloud application to determine the best logistic regression model ( Alban & Mauricio, 2019).

**Table 1.** Classification of data from the dropout student.

Classification	Variable	Description		Source
Personal details	age	Age of the student to the enter the University	numerical	Promotion
	gender	Gender of the student (1 Female, 2 male)	nominal	Promotion
	NSE	socioeconomic level (1 Low, 2 Medium, 3 High)	nominal	Promotion
	VExperienciacInstitucion	Experience with the institution, child of a graduate and graduate (1 Yes, 0 No)	nominal	Promotion
	cve_prospecto	Local, foreign national, and international student (1 Local, 2 Foreign, 3 International)	nominal	Promotion
	cve_city	City of origin (City ID)	nominal	Promotion
Entrance to the University	cve_school	School where the chosen academic program is located (1 Engineering, 2 Administration)	nominal	Scholar
	cve_programa	Chosen academic course (1 to 8 Engineering, 9 to 12 Administration and Business)	nominal	Scholar
	admission	Results of the admission test	numerical	promotion
	verbal	Results of the admission test in the verbal area	numerical	Promotion
	math	Results admission test area mathematical	numerical	Promotion
	wording	Results of the entrance examination area wording	numerical	Promotion
	becaingreso	Amount of scholarship	numeric	Promotion
Academic Data income	average_ingreso	General Average baccalaureate	number	Promotion
	cve_escuelaingreso	School of income (ID numerical)	nominal	Promotion
	type_school	Sector to which the entrance school belongs (2 private or 1 public)	nominal	Promotion
	VExcelenciaacademica	Academic excellence Student with academic excellence greater than 9.5 (1 Yes, 0 No)	nominal	Promotion
	Vaverageacademico	An academic average student with an academic average greater than 9 (1 Yes, 0 No)	nominal	Promotion
	Vsport	Co-curricular sporting experience (1 Yes, 0 No)	nominal	Promotion
Institutional experience	averagefinal	Overall average at the time of exit	numerical	Scholar
	PromedioPeriodoSeleccionado	Average of the last period attended	numerical	Scholar
	Materiascursadas	Total of subjects taken	numerical	Scholar
	Reprobadasarea1	Subjects of the area of general training	numerical	Scholar
	Reprobadasarea2	Subjects of the training area of basic sciences of the school (Administration and Business or Engineering)	numerical	Scholar
	Reprobadasarea3	Subjects of the area of specialization of the academic program	numerical	Scholar
	TotalReprobadas	Total number of failed subjects	numeric	Scholar
	TotalAprobadas	Total number of approved subjects	numeric	Scholar
	Extraordinarios	Total number of extraordinary presented	numerical	Scholar
	lastsemester	Semester in which decides its low	numerical	Scholar
	Becaaverage	The total amount of financial support allocated to the date of its low	numerical	Scholar
	advance	Percentage of advancing the academic program	number	Scholar
	AniosUniversidad	Years student was in the university	number	Scholar
	MotivoBaja	Migration (1), Dropout (0)	nominal	CEDE

The independent variables shown in Table 1 were classified according to what the theorists of attrition observed from pioneering authors such as Tinto (1975), Bean (1985), Spady (1970), Ethington (1990), to some of the most recent authors such as Donoso and Schiefelbein (2007), Manyanga *et al.*, (2017), Aljohani (2016), Morrison and Silverman (2012). In particular, when talking about dropout or retention, the previous authors mention two relevant moments: when the student enters the university and their experience during their stay. As we can see in Table 1, the variables that characterize these moments are categorized in various contexts.

The variable “MotivoBaja” is the dependent variable of this study, while the student’s attributes represent the model’s independent variables. It is worth noticing that the dependent variable points out the reason for attrition, which is a categorical variable. To analyze intercampus migration, we transform this variable into a dichotomous logistic operator that takes values 0 and 1, where 1 represents migration to another campus and 0 any other reason for leaving.

At entering the university, the personal context is composed of the student’s attributes such as age, gender, socioeconomic level, place of origin (foreign or local), and previous relationship with the university. The academic context considers variables related to intellectual attributes. The third context, identified as Institutional, refers to the educational program and the percentage of scholarship granted.

Regarding the second moment, it points out the experience within the institution. Here, we consider variables that explain the academic and financial evolution during the students’ time at the university.

**Figure 3.**
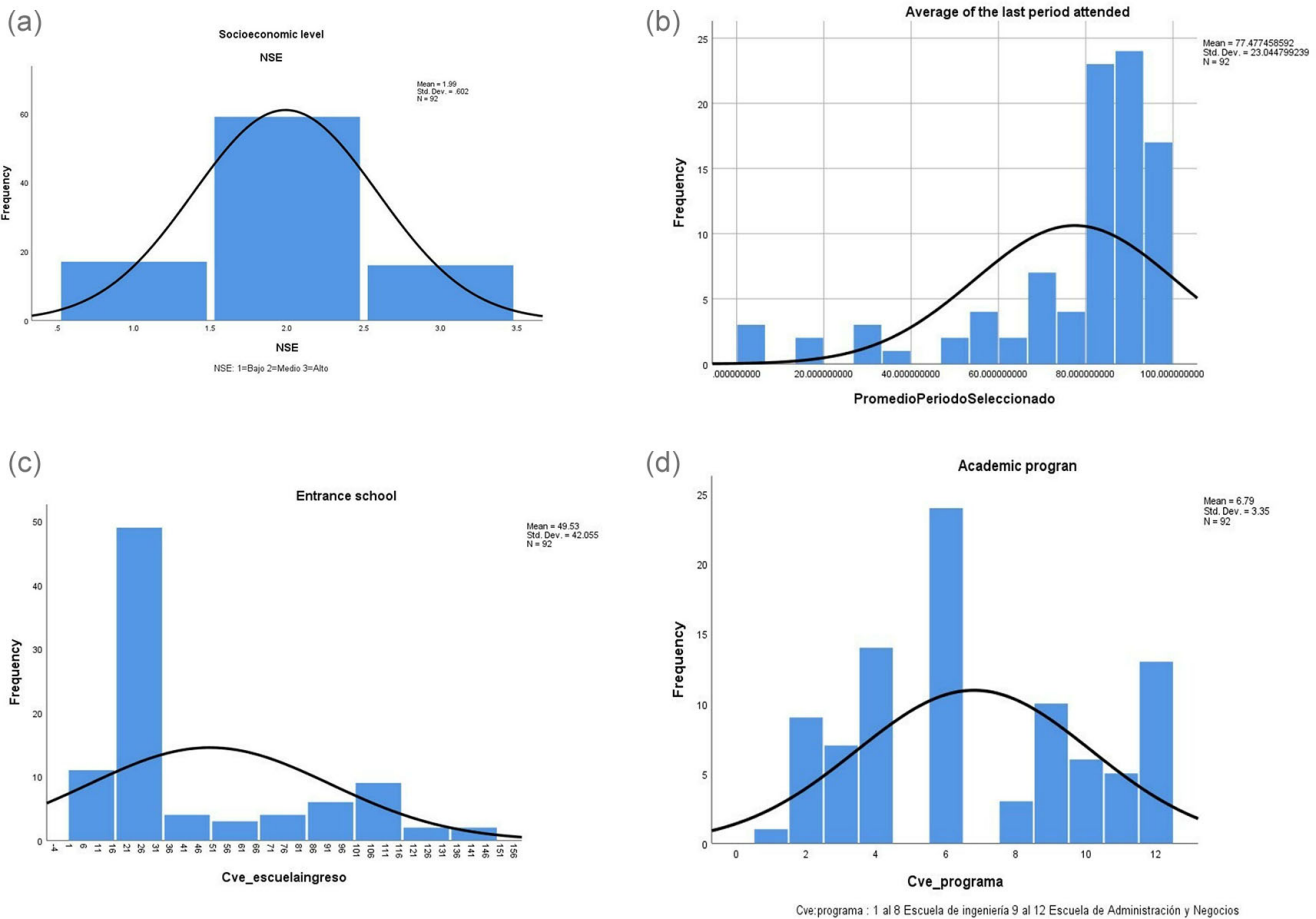
a. Socioeconomic level (IBM SPSS Statistics). b. Final average (IBM SPSS Statistics). c. Entrance school (IBM SPSS Statistics). d. Academic program (IBM SPSS Statistics).

Table 2 shows descriptive statistics of the database we are considering. The dropout students mainly belong to a medium socioeconomic level since the second income quartile is the most common, and many students come from the university’s high school ( Figure 3c). Interestingly, the GPA of migrant students is high; note that the Kurtosis of this variable is positive and equal to three, which means that the distribution is skewed to the right of the mean.

**Table 2.** Descriptive analysis of the most significant variables dropout to other campuses.

		Bachelors’ program	Socioeconomic level	Overall average	School of income
N	Valid	92	92	92	92
	Missing	0	0	0	0
Mean		6.79	1.99	78	49.53
Median		6	2	85	28
Mode		6	2	30a	28
Standard Deviation		3.35	0.602	20	42.055
**Kurtosis**		**-1.22**	**-0.156**	**3**	**2.591**
Standard deviation of Kurtosis		0.498	0.498	0	0.498
Percentiles	25	4	2	73	28
	50	6	2	85	28
	75	10	2	91	81.5

a. Multiples modes. Shows the smallest value.

In addition, we use the IBM SPSS Statistics application, version 25, to perform a descriptive analysis and characterize students’ migration. The variables that show a high concentration of values are the socioeconomic level ( Figure 3a), the final average when dropping out ( Figure 3b), the entrance school ( Figure 3c), and the academic programs per school ( Figure 3d).

We build a mathematical model based on the logistic regression methodology to predict a student’s probability of migrating to another campus. The dependent variable is denoted as *Y*, while the explanatory variables, or independent, are *X1, X2,., Xn.* The dependent variable *Y* refers to students’ migration: *Y* = 1 means that the students drop out from the Ensenada campus to another campus, and *Y* = 0 represents students’ desertion caused by other reasons.

The impact factor of each variable is *β1, β2, …, βn*; and we also consider *β0*, which is the value of the intersection of *Y* when we expect the predictor to be zero. Then, each *β1, β2, …, βn* indicates the slope or adjustment of each independent variable to predict *Y.* If this value is positive or negative, for each unit that is reduced or increased, the closest explanatory variable is predicting the value of *Y.* So, the impact factors capture the relationship between the explanatory variables and the probability of migrating to other campuses within the same educational system. Mathematically, we have that

Y=β0+β1Χ1+β2Χ2+…+βnΧn+ε

where ε is the model’s error.

Using R programming (R Core Team, 2013), we split the database into a training set (75%) and a testing set (25%), which we use to build and validate different models, one for each classification in Table 1. By considering the most significant variables of each model, we construct a fifth model with them. We find that the most relevant explanatory variables of the phenomenon are: age, the result of the admission exam by areas (mathematical, verbal, and writing), the GPA of the student’s last period, the number of failed subjects, how many years the student was in the university and its average scholarship. So, the prediction model that we consider is

model=(age+wording+math+verbal+Reprobadasarea1+PromedioPeriodoSeleccionado+AniosUniversidad+Becaaverage)

## Results

### Predictive model

We construct the best predictive model by adding explanatory variables related to the different dimensions that characterize students’ desertion. Table 3 shows the results of such models that we get using *R programming language*; it is worth recalling that not all variables are significant, so we delete them from the final model. The Akaike information criterion (AIC) indicates that the model that best fits the training data is model 1. The Bayesian Information Criterion (BIC) shows that the confounding variables that impact the decision to migrate to another campus by students are age, the result of the area of mathematics, the grade point average that the student had at the time of migrating, and the scholarship percentage at the time of leaving the campus. Then, the best predictive model is the following:

$$\text{logit}\;\left(1\right)=-1.696-0.109\left(\text{age}\right)+0.002\;\left(\text{math}\right)+0.017\left(\text{Becaaverage}\right)+.017\;\left(\text{PromedioPeriodoSeleccionado}\right)$$

**Table 3.** Comparison of fit between models.

Coefficients	Model 1				Model 2				Model 3			
	Estimate	Standard error	Z Value	Pr(>|z|)	Estimate	Standard error	Z Value	Pr(>|z|)	Estimate	Standard error	Z Value	Pr(>|z|)
(Intercept)	-1.696	1.525	-1.112	0.266	-3.513	2.488	-1.412	0.158	-0.639	1.494	-0.428	0.669
age	-0.109	0.060	-1.819	0.0689.	-0.075	0.063	-1.194	0.233	-0.110	0.061	-1.803	0.07144.
math	0.002	0.001	1.224	0.221	0.002	0.002	1.012	0.312	0.002	0.001	1.545	0.122
PromedioPeriodoSeleccionado	0.017	0.006	2.688	0.0072**	0.017	0.006	2.547	0.0109*				
average_ingreso					0.241	0.223	1.082	0.279				
AniosUniversidad					-0.100	0.114	-0.880	0.379	-0.149	0.111	-1.341	0.180
wording					0.000	0.002	-0.226	0.821				
Reprobadasarea1					-0.053	0.109	-0.491	0.623				
Becaaverage	0.017	0.007	2.410	0.0160*					0.021	0.007	2.915	0.00356**
AIC	**290.78**				**300.06**				**297.27**			
BIC	**308.70**				**328.73**				**315.19**			
Log-Likelihood	**-140.39**				**-142.03**				**-143.63**			
Deviance	**280.78**				**284.06**				**287.27**			
Num. obs.	**266**				**266**				**266**			

**Figure 4.**
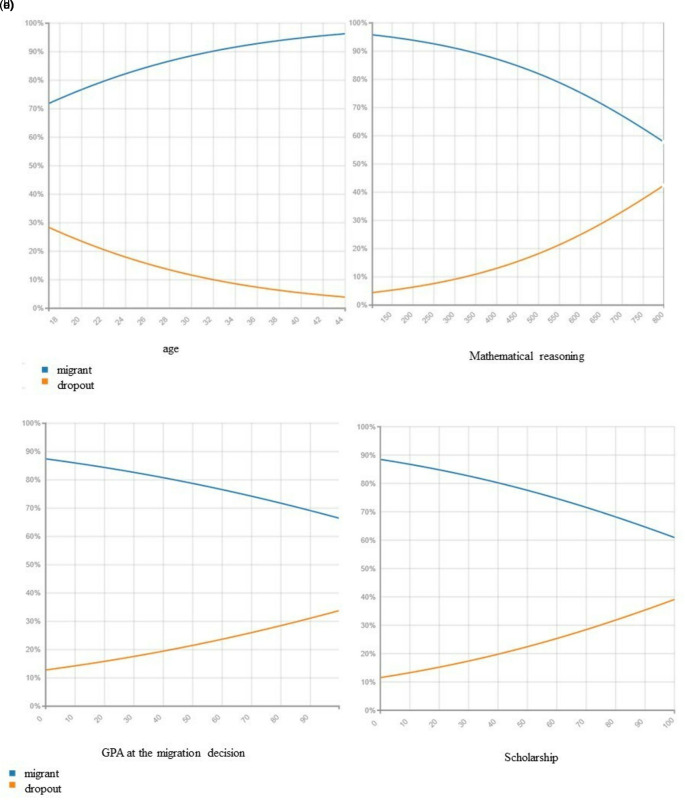
Migration probability given a. Dropout rate, b. Mathematical reasoning results, c. GPA at the migration decision and d. Having a scholarship.

Figure 4a, b, and c visualize the relationship between the decision to migrate among the model variables in. We observe that migration reduces as age increases, but the impact is the opposite concerning dropping out from the system, the older the lower the probability of migrating to another campus but increase the probability of dropping out of the system. However, the probability of migrating to another campus increase as the score on the admission test in mathematical reasoning, the higher the GPA and the scholarship percentage increase.

**Figure 5.**
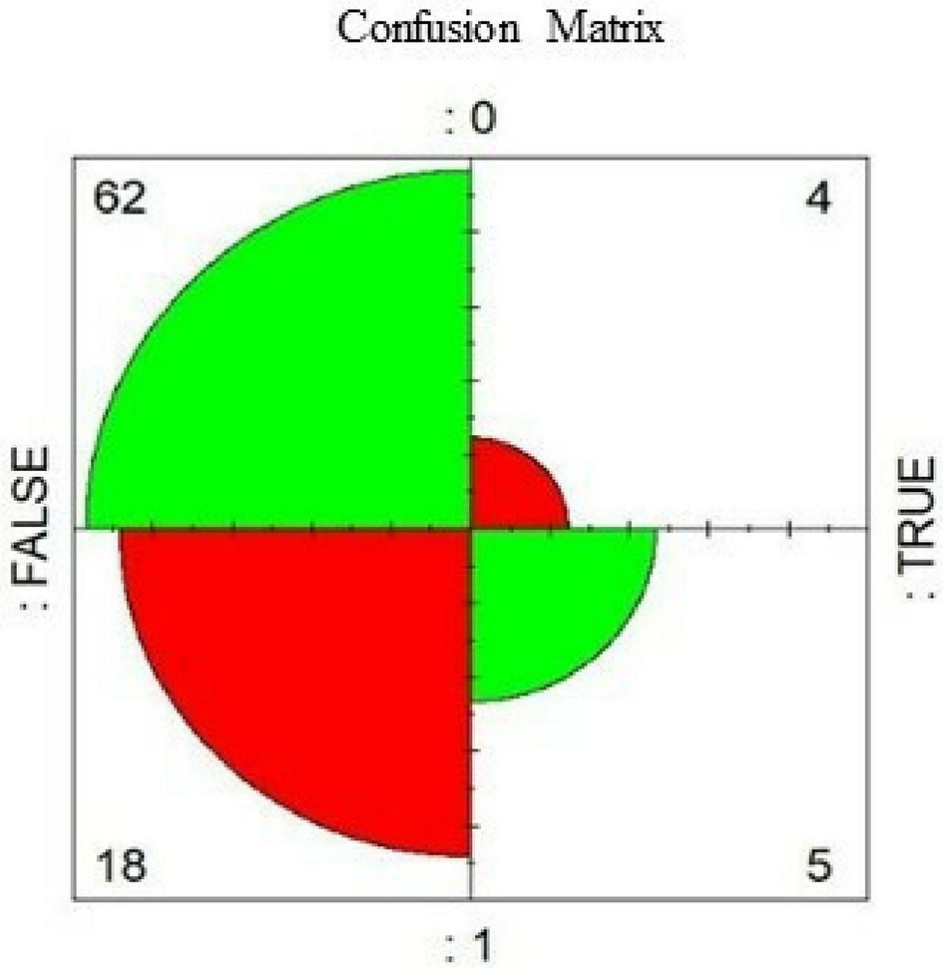
Confusion matrix.

**Figure 6.**
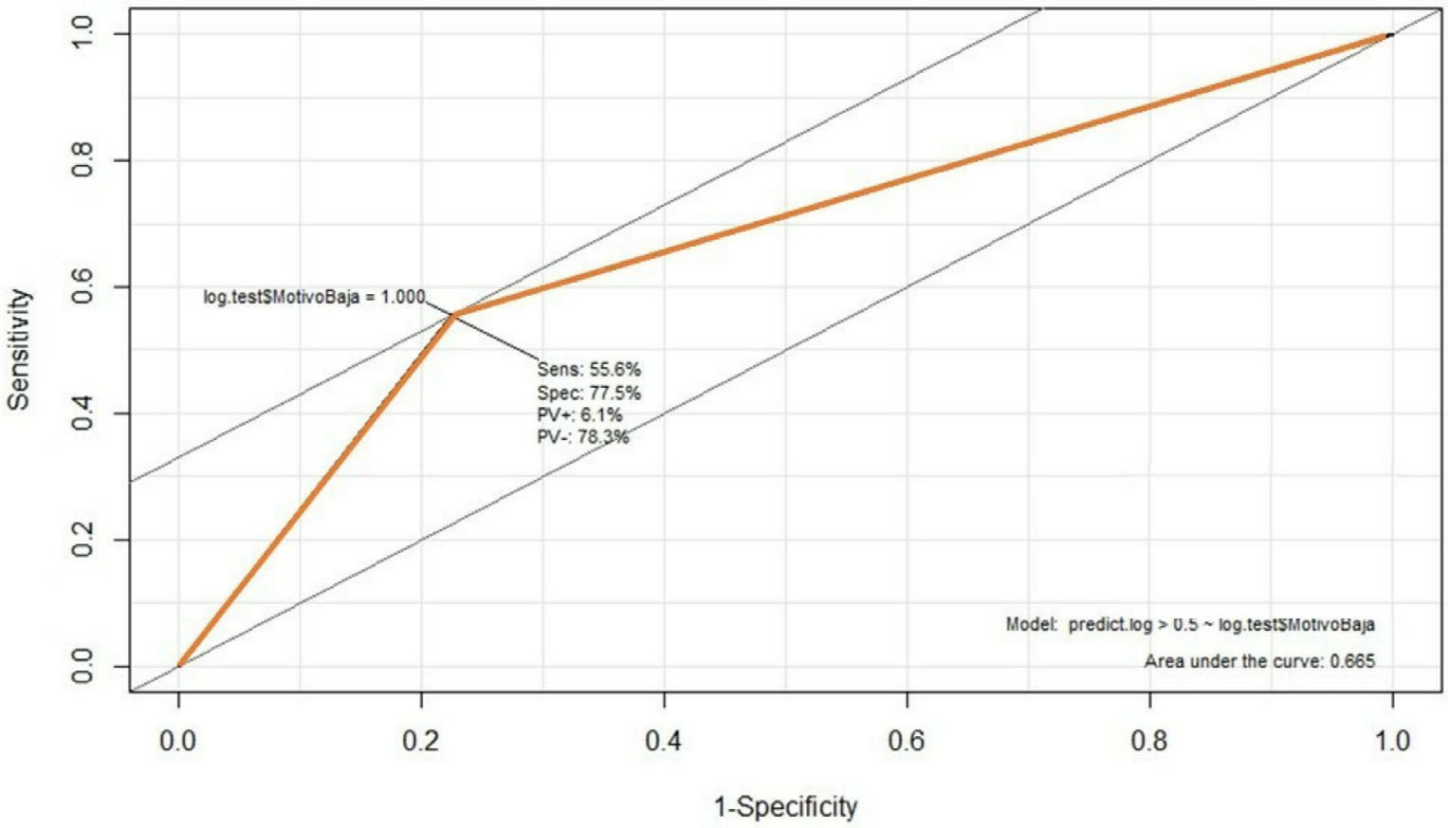
Receiver Operator Characteristic curve (ROC).

Figure 6 illustrates the model assessment concerning its performance, with a 75.28% accuracy (accuracy) and 55.6% precision. The first indicates how close the predicted value is to the actual value, while the second indicator measures how many predicted values are true from all predicted correctly. In addition, the area under the Receiver Operator Characteristic curve (ROC) ( Figure 6) shows the diagnostic capacity of the model with a 67% probability that the prediction is correct. The confusion matrix ( Figure 5) validates the previous results since the model classifies five true positives, 62 true negatives, four false positives, and 18 false negatives, representing a good precision.

We get 77 and 56%, respectively, regarding the model’s specificity and sensitivity. Both metrics indicate the ability of the model to discriminate positive cases from negative ones. In particular, sensitivity measures the positive possibilities, while specificity measures the correct classification of negative instances. The F1 factor of the model is 0.99, indicating a positive relationship between the precision and the ability of the model to predict correctly; this factor is widely used as it suggests the relationship between accuracy and sensitivity, where the model is perfect when F1 = 1. This factor is obtained through the formula:

$$F1=2*\frac{\text{precision}*\text{recall}}{\text{precision}+\text{recall}}=\frac{\mathrm{tp}}{\mathrm{tp}+\frac{1}{2\left(\mathrm{fp}+\mathrm{fn}\right)}},$$

## Discussion

The results allow us to identify the factors that boost students to migrate to another campus. We observe that those with better admission results have a higher probability of moving from Ensenada to other campuses than those with lower results. Moreover, students with a good academic performance, before and during their stay at the institution, are the ones who migrate to another campus. The previous result aligns with the findings of Vera Noriega *et al*. (2012) when they refer to university dropout; their methodology takes up the Astin model that observes entry/exit attributes and the student’s trajectory. Consequently, failure is an important factor for a student to drop out and trust the institution due to the level of commitment they perceive for their permanence. We can conclude that migrant students who continue in the university also trust the institution and its educational model.

Also, Pascarella and Terenzini (1980), Londoño (2013), Peralta, Poblete, and Caro (2017) mention some of the predictive variables concerning dropouts, such as the admission exam and the student’s average when the results are low, which as it could be observed if applies in this case study. However, verbal and writing are not significant, while the opposite happens concerning the results in the mathematical area. You can see that the factor, although it is positive, is very low. It is worth mentioning that these students presented lower writing and verbal results, which is not surprising given their profile. Most of these students belong to engineering programs, see Figure 3d.

Some authors refer to the economic aspect as crucial to determining whether a student chooses to dropout ( Cabrera *et al.*, 1993; Aljohani, 2016). For our case study, the economic variables positively impact the migration of students to other campuses, but they are not significant. We find that the percentage of scholarship favors that students remain in the Ensenada campus.

It is important to note that this model shows that the best-evaluated students in their entrance exam have a higher probability of moving from Ensenada to the other campuses in Tijuana and Mexicali, which is not desirable for the Ensenada campus since we find that the best students are the ones that want to migrate. So, in the long run, such a phenomenon may cause issues concerning the achievement of academic goals that make the campus stand out. For example, the loss of good students diminishes the quality of research projects. It also harms the campus’ retention and terminal efficiency, which impacts the accreditations by study program.

## Conclusions

After grouping all the Ensenada’s dropout students from 2008 to 2018, we observe a large percentage migrating to other campuses. It was possible to identify that these students had a good performance in the admission exam, mainly in the mathematical area; also, these students belong to socioeconomic levels similar to those who dropped out for other reasons, which indicates that the additional support of the campus they may not be having the expected effect in attracting talented students. In addition, all of them obtained some financial support, although these students had the highest scholarship average at their withdrawal. Moreover, our analysis identifies that many of these students come from the university’s high school on the same campus and have a high-grade point average until their last stay on campus.

The previous findings contribute to understanding students’ migration between units or campuses of the same system. However, it is known that the Mexican higher education system consists of 13 subsystems that are classified according to their governing body and their source of education, funding, size, enrollment, specialization, mission, location, and program levels. From 2016 to 2017, around 3,762 Higher Education Institutions offered programs on over 5,000 campuses ( OECD, 2019b). In Mexico, the National Association of Universities and Institutions of Higher Education (ANUIES) points out the existence of 1,228 Higher Education Institutions that belong to a multi-campus system ( ANUIES, 2019). ANUIES counts all Higher Education Institutions; the SEP and ANUIES recognize that many represent a unit set or schools within the same educational system ( Mendoza Rojas, 2018). This research provides valuable information to identify the factors that promote the migration of students between campuses, which may impact the educational system to where they belong. Inter-campus migration is good whenever students get their bachelor’s degree since the country pursues the population’s professional development.

Moreover, it represents the opportunity to have different learning experiences. However, the migration from one campus of the same system means a decrease in the terminal efficiency of the local academic program for the Mexican Accreditation Councils. Then, to avoid inter-campus migration, which can be understood as partial dropout, we should consider an exit survey to measure the students’ perception of the campus’ quality, compared with the one expected from other campuses. Although measurement is not always fast, it is necessary to get more information from migrant students to prevent such a phenomenon.

It is relevant to consider that the most important market for undergraduate programs is the Ensenada campus’ high school. In the summer of 2019, 41% of new students came from the high school located in the same campus ( CETYS University, 2019).

Notice that migrant students can be considered as ‘good students’ since we found that the probability of migration increases as academic variables report high results; then, migrant students trust on the educational system’s quality. Thus, we can infer that migrant students choose to move to other campuses because they want to live the experience of being away from home. In other words, we can infer that the Ensenada campus serves as an intermediate stage before students move to another city. That is to say, parents expect the student to take a few semesters in Ensenada to gain age and maturity and then send him away (to another campus). However, this information cannot be verified as our database does not comprise parents’ information.

Although it is not desertion when undergraduate students move from the Ensenada campus to other campuses, it would be interesting for future research to know if migrating can generate total abandonment. In other words, this paper does not analyze the impact of intercampus migration on the student’s financial health and the campus growth plan. Consequently, this could translate into a decline. Therefore, the administrative and academic departments need to recognize the characteristics of the student who can make this decision over time, understanding that the fixed costs in terms of infrastructure, educational resources, academic staff, service area, and administrative staff are the same. In addition, a higher level of retention is desirable, so analyzing the impact of migration or transfer is essential for future work.

## Data availability

### Underlying data

Zenodo: Desercion a otros campus. https://doi.org/10.5281/zenodo.6377479 ( Beltran, 2022)

This project contains the following underlying data:

- Desercion a otros campus.cvs (migrant students dataset)
  The data variables in the dataset are coded in Spanish; you can see the English description in Table 1.

Data are available under the terms of the Creative Commons Attribution 4.0 International license (CC-BY 4.0).
