# Supplementary material for: Characterizing intercampus migration in a private university of Baja California, Mexico
Source: F1000Res. 2023 Mar 21;11:678. Originally published 2022 Jun 21. [Version 2] doi: 10.12688/f1000research.111079.2 (PMC9918805; doi:10.12688/f1000research.111079.2)
Supplement: Peer review history

# Peer review: Reviewer response for version 1

**DOI:** 10.5256/f1000research.122757.r158431

**Published:** 2023-02-10

**Authors:** Gerardo L. Blanco

**Affiliations:** 1 Educational Leadership & Higher Education, Center for International Higher Education, Boston College, Lynch School of Education & Human Development, Chestnut Hill, MA, USA

This manuscript provides a valuable and timely analysis of important higher education dynamics in the Mexican system. It is based on original empirical research on national context insufficiently studied and therefore, I recommend indexing after minor revisions. 

 I recommend that the authors pay attention to a few minors issues, some concerning with some awkward phrasing:

1. " *Superior education*" is not a phrase employed in the international literature and should be replaced with " *higher education*" or " *postsecondary education.*"
2. While securing ethics approval is often necessary for publication, it is unusual for this to be discussed as extensively as the authors did. I would recommend summarizing the content and eliminating the sub-section. 
3. While the authors do not openly refer to CETYS Universidad as the site of the study, the references to the CETYS website gives it away. I would recommend modifying these references to the website to "institutional website" to protect the institution's identity.
4. The subheading "The data" appears incomplete, and you might consider changing it to "Data and variables"
5. The variables used should be translated to English (e.g., change ' *motivo baja*' with "reason for withdrawal"; age, instead of ' *edad*', etc.).
6. The discussion or conclusion should speak more broadly about what this case can inform higher education institutions in other countries. 

Is the work clearly and accurately presented and does it cite the current literature?

Yes

If applicable, is the statistical analysis and its interpretation appropriate?

Yes

Are all the source data underlying the results available to ensure full reproducibility?

Partly

Is the study design appropriate and is the work technically sound?

Yes

Are the conclusions drawn adequately supported by the results?

Yes

Are sufficient details of methods and analysis provided to allow replication by others?

Partly

Reviewer Expertise:

Higher education

I confirm that I have read this submission and believe that I have an appropriate level of expertise to confirm that it is of an acceptable scientific standard.

# Peer review: Reviewer response for version 1

**DOI:** 10.5256/f1000research.122757.r149415

**Published:** 2022-09-29

**Authors:** Jesabel Gómez Sánchez

**Affiliations:** 1 Universidad Tecnológica del Centro de Veracruz, Mexico City, Mexico

Regarding the citations of current literature, it does not present other works related to the problem of mobility between campuses, they focus on desertion by abandonment. And it does not end up being clear why this would be a problem to society or to students, this seems more beneficial than harmful to them, even what could be a measurement error on the part of universities if these students are not really dropping out, but applying educational mobility.

 On the other hand, the binary logistic regression modeling is shown to be adequate for the approach, supporting the proposed theory.

Is the work clearly and accurately presented and does it cite the current literature?

Partly

If applicable, is the statistical analysis and its interpretation appropriate?

Yes

Are all the source data underlying the results available to ensure full reproducibility?

Yes

Is the study design appropriate and is the work technically sound?

Yes

Are the conclusions drawn adequately supported by the results?

Partly

Are sufficient details of methods and analysis provided to allow replication by others?

Yes

Reviewer Expertise:

economics, econometrics, mainly in business.

I confirm that I have read this submission and believe that I have an appropriate level of expertise to confirm that it is of an acceptable scientific standard.

# Response

**Published:** 2022-10-14

**Authors:** Lucia Beltran

We acknowledge the valuable comment of the referee.

 They highlight an important omission in our work since we did not clearly explain the paper's relationship with the literature. We are aware of the absence of a research paper concerning students migrations since it appears as an exclusive phenomenon of Mexico, given its educational system and the development of Mexican private universities. However, we find that student migration shares features with recruiting students for universities with international branch campuses. In other words, student migration may happen in such a system when students prefer to move from their home university to the international campus. We add to the literature such a discussion. Also, we emphasize the fact that international branch campuses is a recent phenomenon since it is boosted by top universities from the US.
